# Assessing Catastrophes—Dragon‐Kings, Black, and Gray Swans—for Science‐Policy

**DOI:** 10.1002/gch2.201700021

**Published:** 2017-07-28

**Authors:** Paolo F. Ricci, Hua‐Xia Sheng

**Affiliations:** ^1^ University of Bologna (Ravenna Campus) Scienze Ambientali Ravenna 48123 Italy; ^2^ State Key Laboratory of Marine Environmental Science Xiamen University Xiamen 361100 China

**Keywords:** Black and Gray Swans, catastrophic incidents, Dragon‐Kings, nonlinear systems, self‐similarity

## Abstract

The threat of catastrophic incidents—from nonroutine events to extreme ones, such as Dragon‐Kings (DK), Black Swans (BS), and Gray Swans—induces precautionary initiatives that, before the fact, may encounter public resistance or after the fact recriminations. This study develops three aspects of these events: (1) generating mechanisms, (2) the statistical distributions of near and far‐term consequences, and (3) the aggregation of expert opinions about assumptions, mechanisms, and consequences that informs science‐policy. This study shows how causal analysis should account for the: (1) nonlinear catastrophic behaviors that generate predictions, (2) common and power‐law distributions of the consequences, (3) self‐organizing criticality and self‐similarity, and (4) feedbacks and couplings between mechanisms that produce snaps, crackles, and pops as precursor, warning signals. The distribution of the consequences associated with catastrophic incidents has longer and fatter right tails than those expected from failure analysis based on known nonroutine events. DK are extreme events that deviate from these fat tail distributions, have a much higher frequency than expected, and can be predicted unlike BS. This shows how to combine divergent expert individual beliefs over assumptions, causation, and results, and a paradox that affects agreements obtained by majority rule.

## 1 Introduction

This paper unifies salient probabilistic, statistical, and mechanistic aspects of the analysis of catastrophes, providing hands on examples. Science‐policy is informed by the probabilities of adverse catastrophic outcomes (or consequences) per event and their magnitude to gauge the risk reduction of ex ante preventive or precautionary actions, given the model used for prediction. Catastrophes occur far too often to be left without discussions that inform stakeholders about how they occur and thus provide a better understanding of choices that, to save life and property, increase tax burdens, displace individuals, or results in unsightly structures. Unfortunately, the colloquial use of the term catastrophe is ambiguous; a search through the literature yields definitions that range from daily motor vehicle accidents to the Cretaceous‐Paleogene extinction. We use catastrophe as a synonym for the United States Code Service (6 USCS §311):

… “catastrophic incident” [as] any natural disaster, act of terrorism, or other man‐made disaster that results in extraordinary levels of casualties or damage or disruption severely affecting the population … infrastructure, environment, economy, national morale, or government functions ….

We deal with the recent metaphors for catastrophic incidents: Gray Swans (GS),^1^ Black Swans (BS),^2^ and Dragon‐Kings (DK),^3^ and the literature that began with WASH‐1400 (US) and Canvey Island (UK), **Table** **1**:

Typical catastrophic incidents: Nonroutine and Gray Swans (NR, GS). Typical assumption: central limit theorem (CLT), and distributions with finite moments, short‐term correlations in the temporal or spatial data. The mechanisms generating catastrophes are known or knowable and can be modeled by methods such as fault and event trees. Different distributions with either thin or fat tails characterize the output distribution of the physical system, including power‐laws.
Atypical catastrophic incidents: Black Swans. Outliers of unknown origin at the time of their detection. Power laws with fractal exponents characterize their distributions. Scaling equations connect self‐similarity to power laws.
Atypical catastrophic incidents: DK. Self‐organizing criticalities (SOC) can be unpredictable when observed at the micro or macro scales. An emergent new order arises from correlations at different time and space intervals: couplings become synchronized. SOC may be on the verge of a chaotic change: cascading events, sustained drift, or abrupt endogenous shocks can cause *Dragon‐Kings*.

**Table 1:** Summary characteristics of Nonroutine, Black Swans, Gray Swans, and Dragon‐Kings events (motivated by Kovalenko and Sornette, 2013^64^)

Catastrophic Incidents					
Event → Key↓ Characteristics	Nonroutine (NR)	Black Swans (BS)	Gray Swans (GS)	Dragon‐Kings (DK)	Notes
Distribution of consequences, given the class of catastrophic incident	Rapid convergence to a finite maximum consequence	Slow convergence to a maximum consequence	Slow convergence to a maximum consequence	Jump in the probability of a very large consequence relative to the distribution	Focus on consequences ranging from physical to financial measured by a variety of metrics.
Theoretical predictability	High	Lowest, no warnings	Medium, available theory	High, with early warnings	Relative to NR
Empirical predictability	High	Lowest, the past does not predict the future	Medium, available historical data	High	Relative to NR
Impacts magnitude	From Large to Extremea)				None
Formal justification of ex ante precautionary policies	Can be related to cost effectiveness or risk‐cost‐benefit (RCB) analysis	Cannot be related to RCB analysis but can be used in risk‐risk assessment	Can be based on risk‐cost‐benefit (RCB) analysis or cost effectiveness	May be related to risk‐cost‐benefit (RCB) analysis or cost effectiveness	Policies may not be practically implementable due to the extreme magnitude of the instantaneous consequences

^a)^ Includes international collateral damage or effects such as contagion. Internal and external forces (such as shear, pressure, heat and so on) change the state of a system from equilibrium to disequilibrium; may reduce system's resilience and amplify its vulnerability.

**Figure 1:**
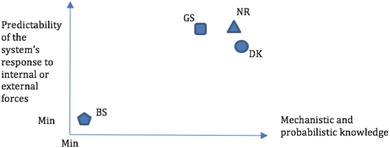
Relative level of probabilistic knowledge and predictability of response of the main catastrophic events discussed in this paper.

**Figure** **1** summarizes the relative predictability versus knowledge (Figure 1; BS is Black Swans, GS is Gray Swans, NR is nonroutine, DK is Dragon‐Kings) to orient the readers.

**Figure 2:**
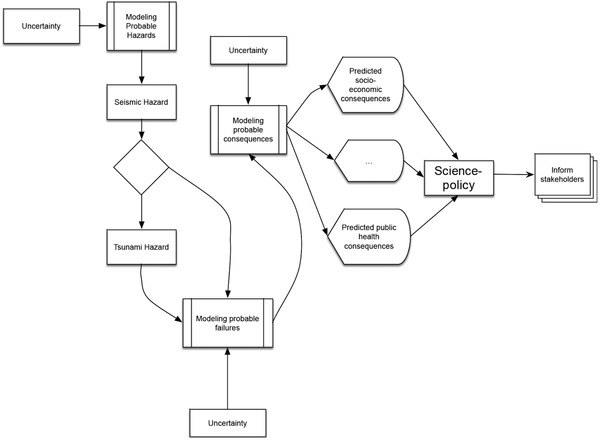
Simplified flow of information regarding the possible effects of a hypothetical earthquake‐tsunami coupled or decoupled event.

Catastrophic incidents leading to large or extreme adverse consequences are increasingly associated with natural causes; their consequences are often made worse by overcrowding, poverty, and other socio‐economic factors. The analysis and prediction of those consequences^5^ requires combining mechanistic processes with mathematical, statistical and probabilistic models, **Figure** **2**.

The probability of a catastrophic incident requires formal analyses to account for the temporal and spatial distributions of its multiple consequences. This accounting often combines with majority or consensus‐based scientific opinions and results in costly, but certain, preventive or precautionary actions. Yet, when built, those costly choices may appear not serve their purpose because the average arrival time of the catastrophe (i.e., human‐made, natural, or a hybrid of these two) is often much longer than the lifetime of those making the decision to act preventively. To account for these eventualities, analysts tasked with informing stakeholders consider:

1. Different spatial and temporal changes in the consequences.
2. The probability that catastrophic incidents occur, perhaps in clusters.
3. Short‐ and long‐term actions that can partially dominate each other: dominance may reverse over time due to unexpected endogenous or exogenous events.
4. Inability to calculate the values of sample information and flexibility because of the length of the inter‐arrival times cannot account for technological advances.
5. Microscopic behaviors that, although individually unlikely to cause harm, in the aggregate result in a catastrophic incident.
6. Emergent behaviors for which knowledge of one or more sub‐systems does not allow predicting the entire system's behavior.
7. Data and process complexities (e.g., signal to noise ratio; type of noise (white, red, etc.), short and long‐term correlations, uncertainty about causation, as risk factors and processes, the effects of nonlinearity).
8. Distribution of consequences over those at risk, their frailty, the social cost of preventive actions, the type and magnitude of the direct, indirect, and unintended consequences after the catastrophe.^6^

Thus, informing science‐policy reflects aspects of the:

perceptions of the reality associated with the catastrophe of concern, the state of information and knowledge, inadequate or incomplete data, failure of imagination, biases, ambiguity, ignorance, …, decision‐makers' and stakeholders' attitudes towards risks of loss, …, limited number of scenarios selected by consensus, …, incorrect or not trusted communications, …, ideology, …, and so on.

**Figure 3:**
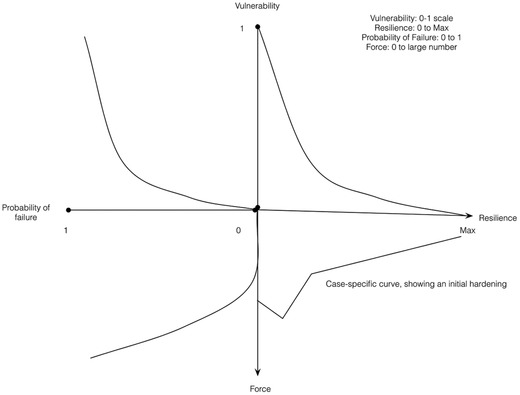
Relationships between a physical system's vulnerability, resilience, force (shear, pressure, strain, etc.), and probability of total failure (motivated by Kovalenko and Sornette, 2013^63^).

**Figure** **3** depicts an idealized set of relationships based on a system's vulnerability, resilience, forces, and probability of failure. The four relationships in this Figure are case specific; we do not bound resilience because a system may have some residual direct use, but we bound vulnerability as it implies complete operational failure at 1.0.

## 2 A Key Policy Aspect of Catastrophes

We begin with policy dilemma exemplified by the possibility of very large slope failures from flank instabilities in Hawaii likely to affect New Zealand^7^: Huge sector collapses (1000–5000 km^3^) … on the flanks of Hawaii volcanoes… have been modeled to produce Pacific‐wide tsunamis … of hundreds of meters. The dilemma is dismissing the catastrophe—knowing that it may destroy a large part of New Zealand—because:

Their return periods from any one source are well in excess of the returns … of interest in this study. Therefore, no landslides at global distances are considered viable tsunamis sources within the 2500‐year period of this risk study

The average return period removes danger. Yet, although the return period is an important descriptive statistical quantity, it is a questionable metric when dealing with events with large consequences. If the return period of a catastrophe is 100 years, assuming a binomial distribution, the expected frequency of that event is p = 1/100 = 0.01. Yet another relevant question policy question is: What is the probability that the 1 in 100 years' event occurs exactly once in ten successive years? The binomial distribution function: *f*(*k*; *n*, *p*) = *_n_C_k_ p^k^*(1 − *p*)*^n^* ^−^ *^k^* accounts for all possible sequences of successes or failures; *k* is the number of successful outcomes out of *n* total outcomes, (*n* − *k*) are not successful. Hence: _10_ *C*_1_ × 0.01^1^ × 0.99^9^ = 10 × 0.01 × 0.914 = 0.0914: about a 9% chance of occurring.

Regarding the spatial domain, the USGS earthquake probability mapping calculator (geohazards.usgs.gov; accessed September 4, 2015), for major earthquakes (magnitude: *M* > 8.0 within 100 years and 20 years, respectively; the USGS uses the moment‐magnitude scale, *M*_m_) and for relatively minor earthquake (magnitude *M* > 5.0, within 20 years), yields areal probability‐magnitude relationships and thus extends the frequency–magnitude (*F*–*M*) representation.

An empirical distribution of seismic moments (*M*_0_), exemplified and discussed by Sornette as a power law distribution, for earthquakes in California, is: (log number of events) = (*a* − 2/3log(*M*_0_)).^3^ This curve is linear in the log–log space and fits the data between *M*_0_ = 24 to 27 very well; but is increasingly less accurate thereafter. The relationship between magnitude (Richter scale), level of damage, and amplified ground motion (in microns) can be found in ref. 4.

The number of prompt deaths from worldwide earthquakes, from 1900 to 2011 (Jorgustin, 2011), captures the impact of these catastrophes. The highest, approximate number of deaths occurred in China in 1976 (300 000 deaths), in Sumatra in 2005 (230 000 deaths), Haiti in 2011 (230 000 deaths) and in China in 1921 (200 000 deaths).^8^

### 2.1 Probabilistic Representation of Routine and Nonroutine Events

**Figure 4:**
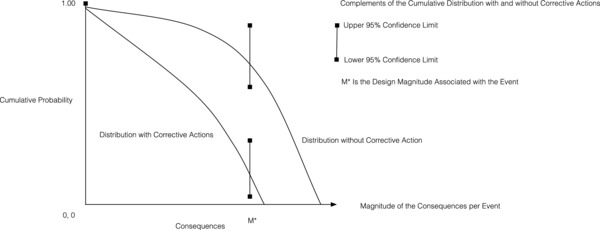
Hypothetical 1D cumulative frequency‐magnitude *F*–*M*, functions for the consequences from a single event, with and a without corrective choice (data to which the curves are fit not shown).

Deaths are but one of the many consequences from a catastrophe. If an underwater earthquake generates a tsunami, the initial wave may start by being 40 cm in height and its period several hundred kilometers in length. At the shoreline, however, the waves may be between 20 and 30 m high and have a period of 1–2 km. The probability of the tsunami occurring cannot be changed. What may be changed is the risk profile of the consequences associated with the arrival of a tsunami of a specific height at a specific shoreline location. Suppose that decision‐makers consider two options: do noting versus take corrective action by building a 30 m sea wall of an appropriate length based on locality conditions. We represent these alternatives by the complements of the cumulative frequency–magnitude distribution functions, **Figure** **4**, depicting the probability density function^9^ of the random variable number of prompt deaths, *M*. Other outcomes, e.g., property destroyed, can be depicted similarly.

Each curve is a conditional expected value; the confidence limits shown at magnitude M* describe the variability of the data to which a curve is fit at that point. Hence, the vertical bar represents the uncertainty bound for that magnitude, conditional on historical data to which the curve is fit. For simplicity, we do not show the confidence lines about each of these curves, as should be done in practice. These curves may not be statistically distinguishable in the frequency–magnitude space: this would be depicted by overlapping uncertainty bounds. In practice, a protective wall can prevent deaths from locally smaller waves, while somewhere else along the same seawall, larger waves may overtop it. This probabilistic characterization may extend to accounting for the^10^:

1. Magnitude of the design event (e.g., as discussed next, the Fukushima (Tohoku's) earthquake seismic moment (M_o_) was 3.9 × 10^22^ joules; the surface energy of the seismic waves was 1.9 × 10^17^ J (USGS, Oct. 24, 2012; earthquake.usgs.gov)).
2. Unbiased assessment of the historical record of events leading to similar catastrophes locally.
3. Possible risk factors likely to magnify the magnitude of the losses (e.g., high population density new buildings along a shoreline, time of day occurrence of the event, etc.).
4. Number and types of individuals at risk (e.g., vulnerable populations).
5. Stock and location of property at risk, including sensitive public and private installations and key infrastructure.
6. Probability, magnitude, and severity of collateral consequences.
7. Severity of direct and indirect damages to inhabitants, workers, property, and so on.

Elements 1 through to 7 inform actions such as:

8. Ex‐post mitigation of damage through emergency actions, short‐ and long‐term reconstruction, and relocations.
9. Ex ante mitigations (such as strengthening critical infrastructure, high density buildings, and so on) at several scales.
10. Public health interventions for possible increased disease burdens generated by NR‐type events due to water, sanitation, and other infrastructure failures.

Catastrophes also cause environmental, ecological, social, psychological, and cultural damages. These may be assessed with different methods than those we describe in this paper, with damages measured by loss of species habitat, stress, loss of cultural artifacts, and many others.

### 2.2 Science‐Policy Dilemma

The large‐scale collapse of sides of a Hawaiian volcano are beyond human control. However, smaller catastrophes may be foreseeable and controllable but are probabilistic regarding the event itself and the consequences. Decision‐makers will generally face a dilemma. Suppose that at shoreline specific locations the following alternatives are plausible from an engineering view:

1. Use an event with a 1/100 return period, with a 10% probability of occurring in the next 10 years and build a 10 m average height seawall) of a specific length (e.g., 200 km) or
2. Use a more infrequent event (e.g., 1/500 year with a 2% chance of occurring over the next ten years) wave height and build a 30 m (average height) wall (e.g., 400 km) or
3. Do nothing, with certainty.

The dilemma suggests a science‐policy solution through a Bayesian probabilistic decision model (e.g., conditioned on a specific event such as a magnitude 8.5 off‐shore earthquake). The analysis would yield: the expected values of the consequences (e.g., average number of prompt deaths), the values of information and flexibility associated with the cost of obtaining additional information and developing flexible alternatives. The criterion for justifying the best option might be to minimize the expected number of deaths associated with each alternative.^11^ The approach accounts for: (i) new knowledge becoming available at time intervals much smaller that the return periods of the event of concern, and (ii) although initial capital costs may be higher, the overall costs of the preventive action may be significantly smaller. The initially large expenditure allows flexibility for later design changes, either up or down, as new information becomes available.

### 2.3 Examples: Japan's Tohoku (2011) and Nepal's Gorkha (2015) Earthquakes

Japan began protective measures, developed after the 1933 Showa earthquake that generated a 29 m (maximum height) tsunami, in the same region as Tohoku's earthquake of 2011. Those included relocations, coastal dikes, tsunami control forests, seawalls, tsunami‐resistant areas, evacuation routes, tsunami watches, evacuation, and memorial events. The Tohoku earthquake was a 1/1000‐year event with a tsunami with a maximum wave height of ≈41 m. The Smithsonian reports of a local effect of the tsunami^12^:

… the peak of the 2011 tsunami reached 138 feet high …. When the wave reached Otsuchi, a town of 15 000 people, it was 50 feet high. It easily breached the town's 30‐foot wall. Up the coast, the town of Fudai was barely touched

It caused 15 891 deaths, 6152 injured, and displaced ≈230 000 persons. Property damage included ≈130 000 destroyed, 270 000 partially destroyed, and 750 000 partially damaged buildings (Japanese National Police Agency Report, February 10, 2014). As reported by The Economist^13^:

A few months after the disaster it pledged to build hundreds of seawalls and breakers in the three worst‐hit prefectures of Fukushima, Miyagi and Iwate. The total cost will be up to ¥1 trillion ($9.8 billion). More walls are planned. A report by the ministries of agriculture and land said 14 000 km of Japan's 35 000 km coastline requires tsunami protection. Seawalls are controversial. They look hideous and the evidence for their effectiveness is flimsy. True, Fudai, a village sheltering behind a giant concrete shield, escaped unscathed in 2011. But in the city of Kamaishi a $1.6 billion breakwater, listed in the “Guinness Book of Records” as the world's largest, crumbled on impact. Nearly 90% of existing seawalls along the northeast coast suffered a similar fate. Critics say they even resulted in greater damage being caused elsewhere. “There is simply no guarantee that seawalls will stop every single tsunami,” says Nobuo Shuto, an engineer at Tohoku University.

Ex ante for the Tohoku earthquake, the option do not build a seawall is relevant because a tsunami wall had been constructed before 2011.^14^ As The Economist concluded^13^ regarding its success as a preventive measure:

Even more puzzlingly, the land ministry admits the new structures are not designed to withstand the sort of seismic event that occurred in 2011. That earthquake is considered a once‐in‐a‐thousand‐years calamity and nothing could block it, says a spokesperson for the ministry. Koizumi's wall is less than half the size of the highest wave that hit the area three years ago. The walls may even make things worse. The 2011 deluge killed Ms Otsuka's mother and her brother's two children. They could have been saved if they had fled 10 m up a hill behind their house, she insists. They didn't run because they thought the seawall would protect them.

The Institute for International Studies and Training of Japan^15^ estimated macroeconomic losses from the Tohoku events (namely tsunami, earthquake, and nuclear incident):

Massive efforts by those involved have achieved solid progress toward disaster rehabilitation, and the Tohoku economy is gradually recovering from its post‐disaster slump, with a steady overall recovery underway. According to macro data, the industrial production index for the Tohoku economy slumped from 99.7 (topping the national index of 98.5) in February 2011 immediately before the earthquake to 64.2 in the March immediately after, well down on the national index of 82.5. However, by March 2012, a year later, Tohoku's industrial production had returned to the same level as the rest of the country.

The World Bank calculated that the economic cost of the Tohoku earthquake was as high as USD 235 billions, ≈4.1% of Japan's GDP (The Economist, Counting the costs, March 21, 2011; accessed February 7, 2017). In 2011, Japan's per capita income was USD 46 200; its GDP was ≈6.157 trillion USD (The World Bank, Japan Economic data, accessed February 7, 2017). Industrial production was also affected rebounding relatively rapidly.^15^ As a baseline, in 2014, Japan's GPD was 4.849 trillion USD (The World Bank, Japan Economic data, accessed February 7, 2017); its population was ≈127 million, with a per capita GDP of 36 194 USD (The World Bank, Japan Economic Data, accessed February 7, 2017); life expectancy at birth was 83 years. Mortality rate for the under 5 years old was 3 per 1000 live births. The drop in the Japanese industrial production index due to the Tohoku earthquake and tsunami at the local and national levels and its rapid rebound.^14^

The World Bank also describes aspects of social dislocation from both tsunami and earthquake damage to four reactors (of which three were core meltdown accidents) of the Fukushima‐Daiichi (Fukushima one) nuclear power plant, which consisted of six boiling water reactors (BWRs) units with an overall combined electric power generation of about 4.7 gigawatts electric. At the time of the tsunami, three reactors were not operational for refueling and maintenance.

A more recent earthquake of magnitude (*M*_s_) 8.1 in Nepal, in April 2015, the Gorkha earthquake, caused at least 9000 deaths and more than 22 000 injured and economic losses of ≈35% of Nepal's GDP (about USD 20 billions in 2012). Nepal's GDP in 2014 was 19 636 million USD; its per capita GDP was ≈698 USD in 2014 (The World Bank, accessed August 5, 2015); life expectancy at birth was 68 years (in 2013), with a mortality rate of 40 per 1000 live births in those under 5 years. These economic and social data are an example of what may be initial conditions regarding the overall effect of major catastrophes on a country, and its potential and rates of coping with fatalities, injuries, destruction and the potential for rebounding. For Nepal, the rebounding has been much slower than for Japan: a year after the earthquake there is no signs of any rebuilding.^16^

### 2.4 Examples of Other Catastrophic Incidents

Earthquakes and tsunamis are not the sole causes of catastrophes beyond human control.^17^ Extremely large natural catastrophes include asteroids' and comets' impacts, and super volcano eruptions (magnitude ≥ VEI 8, Volcano Explosivity Index). The latter produce deposits greater than 1000 km^3^ (>240 cubic miles). Instances of those eruptions include: Yellowstone (Wyoming, USA), Long Valley (California, USA), Toba (Indonesia), and Taupo (New Zealand). Toba's eruption, about 74 000 years ago, destroyed more than 99% of the human population (apparently reducing it from 60 000 000 to < 10 000, although these numbers are disputed). The most recent super volcano eruption occurred 27 000 years ago at Taupo, in New Zealand. Currently, Italy and other countries including the US may be under threat. The Yellowstone super volcano is an example. As the USGS states^18^:

Although it is possible, scientists are not convinced that there will ever be another catastrophic eruption at Yellowstone. Given Yellowstone's past history, the yearly probability of another caldera‐forming eruption could be calculated as 1 in 730 000 or 0.00014%. However, this number is based simply on averaging the two intervals between the three major past eruptions at Yellowstone – this is hardly enough to make a critical judgment. This probability is roughly similar to that of a large (1 km) asteroid hitting the Earth.

And yet^19^ states that:

Now researchers report that the source beneath the surface may be even more massive than previously thought…., they have created an image of the plume beneath Yellowstone showing the cyclone shape stretching at a 40° angle to the west at a depth of 200 miles for 400 miles east to west, as far as the new technique can reach. … The study does not make … predictions about future eruptions, which the USGS Yellowstone Volcano Observatory notes are of very low probability in any given millennium, since they have been separated in the past by 800 000 and 660 000 years.

The Yellowstone event would be unprecedented for the United States, although the overall effects are disputed.

### 2.5 Policy Comment

Science‐policy informs stakeholders and decision‐makers through describing and predicting the societal value of taking immediate or phasing preventive and precautionary actions. Developing the appropriate portfolio of choices, selecting and implementing those that minimize or prevent adverse consequences at the least cost raises these threshold questions:

1. What is the set of consequences (by type, time, space, severity, etc.) of policy concern?
2. How are the consequences distributed on those at risk, and what is the locality of the catastrophic incident?
3. What magnitude of the consequences triggers ex ante preventive and corrective actions?
4. Should both probability and magnitude thresholds mobilize the ex ante allocation of resources and corrective or preventive actions?

## 3 Black Swans, Gray Swans, and Dragon‐Kings

In the context of these catastrophic incidents, large magnitude low probability consequences are fit to power laws, rather than the standard distribution with very thin right and left tails. For example, a power law is, for sufficiently large values of *x*: *p*(*x*) ∝*c_μ_*/*x* ^1+^ *^μ^*, which generates heavy right tail for μ < 2.0.^3^

### 3.1 Black Swans

A BS does not produce precursor warnings: its salient policy characteristic is surprise and large magnitude. It produces a confirmation bias: many high frequency low magnitude consequences condition stakeholders to expect another similar low magnitude event: the BS is dismissed as unpredictable.

The … expression derives from the Old World presumption that all swans must be white because all historical records of swans reported that they had white feathers. … a Black Swan was impossible or at least nonexistent. After … Willem de Vlamingh … in 1697, discovered Black Swans in Western Australia,^20^ the term metamorphosed to connote that a perceived impossibility might later be disproven.

Indeed:

… Black Swan was a Latin expression–‐its oldest known reference comes from the (satiricist) … Juvenal's characterization of … being ‘rara avis in terris nigroque simillima cygno' (who was actually satirizing women…).^20^ … Latin phrase means ‘a rare bird in the lands and very … (similar to) a Black Swan'.

Taleb states^2^:

… a Black Swan … is an event with the following three attributes. First, it is an outlier, as it lies outside the realm of regular expectations, because nothing in the past can convincingly point to its possibility. Second, it carries an extreme impact. Third, in spite of its outlier status, human nature makes us concoct explanations for its occurrence after the fact, making it explainable and predictable.

Before their discovery, could Black Swans be conjectured to exist? In Europe evidence of changes in bird plumage color due to their diet was known before 1697, (e.g., flamingos). This fact can produce a biological conjecture—Black Swans exist but have yet not been found—that has a mechanistic basis and is qualitatively different from an outlier. Black Swans are observed and obtain from an undisclosed underlying physical mechanism that yields outcomes modeled by the stretched negative exponential distribution. Black Swans are much rarer and of larger magnitude than those generated by the exponential distribution.^21^ Outliers are observed empirical values due to errors, chance, or other reasons, including new mechanisms: they inform from common errors to new phenomena. Continuing with the metaphor, consider the weight of white swans. Very low frequency, very heavy white swans are Black Swans within a sample of white swans. When these swans cluster, they might signal a change in the population from several possible causes. These include different diet, changes to the cluster's ecosystem, or a random genetic event. Regarding the extreme impact, white swans of very heavy weight may place upward pressure on existing population of white swans.

### 3.2 Gray Swans

Lin and Emanuel (2015) empirically define Gray Swans as tropical cyclones that produce high‐impact storms.^1^ Specifically, these would not be predicted based on history but may be foreseeable using physical knowledge together with historical data. While lack of empirical evidence to support a theory is well known in the sciences (e.g., factual confirmation of a theory may take decades), it is a critical aspect of precautionary policy‐making. These authors state that:

Some high‐consequence events that are unobserved and unanticipated may nevertheless be predictable (although perhaps with large uncertainty); such events may be referred to as ‘Gray Swans' (or, sometimes, ‘perfect storms'). … Gray Swans—although also novel and outside experience—can be better foreseen and system‐atically prepared for. Prediction of a Gray Swan … is meaningful and practically useful only when associated with some likelihood/probabilistic statement; for example, the probability of exceeding the storm surge level induced by the Tropical Cyclones in a year is 10^−3^.

Lin and Emanuel (2015) use statistical‐deterministic climatological‐hydrodynamic mechanistic models to identify storm surges from tropical cyclones (TC) for several areas of the world^1^, ^22^ Importantly, by adding events such as climate change, they find that the combined magnitude of the storm surges generated by Gray Swans can be extreme (e.g., from about 7 m without climate change effects to 11 m in Tampa, Florida). For instance, the probabilities can increase significantly over the twenty‐first century (to 1/3100–1/1100 in the middle and 1/2500–1/700 toward the end of the century). More specifically:

… Gray Swan TCs as the synthetic TCs that are associated with extremely low annual exceedance probabilities (large mean return periods) of the induced storm surges tropical cyclones have been limited by the comparatively short length of historical records. This limitation is being overcome by the new field of palaeo‐tempestology, which identifies TC events in the geologic record, and by bringing knowledge of storm physics to bear on the problem.

The probabilistic framework of Gray Swans consists of physical models (e.g., Newtonian deterministic physical laws) with stochastic processes (e.g., Brownian, Markovian, Poisson).

### 3.3 Dragon‐Kings

**Figure 5:**
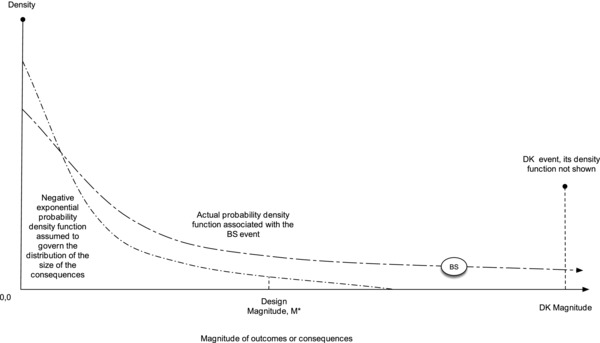
Hypothetical empirical fat‐tail and exponential distributions for: nonroutine event and hypothetical design magnitude M*. Black Swan power law is a heavy‐tailed distribution relative to the exponential. A BS event is an unexpectedly high magnitude‐frequency point that belongs (but is not known to belong) to the fat tail distribution: It is an outlier after the fact. The Dragon‐Kings event is even higher magnitude and frequency (distribution not shown) than the BS.

**Figure** **5** depicts a DK event, a Black Swan outcome, and the more common exponential distribution for a specific event and a design magnitude M*. (new.scientist.com, accessed July 30, 2015). Sornette (2009) defines as the existence of transient organization into extreme events that are statistically and mechanistically different from the rest of their smaller siblings.^3^ Those siblings belong to the power law distribution—its data and their statistical analysis fit a power law distribution and thus have a common generating mechanism; predictability is minimized because the same generating mechanism operates over several orders of magnitude of the consequences. A DK has different probabilistic, statistical, and mechanistic basis: these make the DK event predictable.^23^ For instance, phase transitions, bifurcations, and tipping points, with emergent organization that produces useful precursors to the DK.^3^

**Figure 6:**
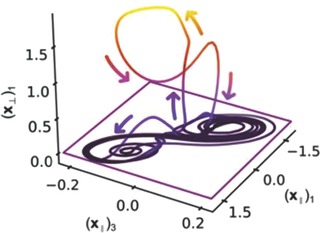
Experimental observation of attractor bubbling in a coupled chaotic oscillator.

In Figure 5, the BS event is wholly unexpected when the exponential distribution is incorrectly assumed; M* is expected and drives protective policy. The DK is an event that does not belong to either of these two distributions, as Figure 5 depicts, because it is generated by a change in the behavior of the process that produces the data to which the distribution curve is fitted. If we take the fat tailed distribution, the DK is characterized by a sharp jump in the frequency at the DK magnitude. The jump may be immediately preceded by a dip in the data: a warning of the impending catastrophe that is not common to either the BS or the M* values (Figure 5). The dip at the low frequencies that immediately precede the DK is also unlike higher frequencies, low magnitude fluctuations: it presages a new mechanism. For example, Sornette (2002) studied large changes in stock market indices finding that bubbling regimens precede them.^24^ Johansen and Sornette state that (for runs of losses) about 99% of them follow an exponential distribution with a fat tail and study several more financial mechanisms and their DK behaviors,^25^ including crashes.^26^ DKs behavior include bubbling or riddled basins of attraction or rejection.^27^ The system generating them, coupled oscillators, may exhibit trajectories that occasionally result in outcomes of large magnitude and probability; **Figure** **6** (modified from Cavalcante) shows bubbling, as a transient relative to the system's state. The DK event is a jump in a trajectory away from the invariant manifold (the set of solutions generated by different initial conditions) and return to these. It does not belong to the scale‐free distribution, is distinguishable from the time series of data, and controllable. Coupled oscillators include earthquakes, some financial systems, and saturation mechanisms.^28^ The DK in Figure 5 is not an outlier^29^ in the sense of a BS because it is generated by known coupled differential equations with initial conditions. The prediction from the power law would give the magnitude of the DK a much lower probability (Figure 6).

For instance, a DK may be associated with the characteristic earthquake hypothesis, which occurs when coupling is high and heterogeneity low.^30^ The physical system that generates the DKs can be perturbed to better study it.^23^, ^31^ Large forces may not cause catastrophes but amplification by faulty cooperative mechanisms does. Exogenous stresses are also important: once the system reaches an unstable equilibrium it is at a critical point; a small force breaks the proverbial camel's back. A signature for that event, such as bubbling, initiates with hot spots within the chaotic attractor.^30^ Log‐periodic oscillations describe the behavior of a bubble over time. Sornette, Johansen, and Bouchaud introduced a model to predict events such as earthquakes and financial market crashes using a log‐periodic power law.^26^ Brée, Challet, and Peirano give comprehensive review of the issues associated with fitting log‐periodic functions to noisy time series.^32^ Based on self‐reinforcing behaviors and risk premium for remaining invested, a model to predict singularities (i.e., catastrophic incidents) and fitting the model to the data^26^, ^33^ is: *y*(*t*) = *a* + *b*(*t_c_* − *t*)*^z^* + *c*(*t_c_* − *t*)*^z^* [1 + *c*cos(ωlog (*t_c_* − *t*) + φ)]. In this model: *t_c_* is the most probable time of the crash, *z* the growth exponent, ω controls the amplitude of the oscillations; *a*, *b*, *c*, and φ are parameters to be estimated from the data. For a rapidly accelerating value of *y*, when *t* approaches *t_c_*, the oscillations have higher frequency and decreasing amplitude. These characteristics suggest that it may be possible to identify signatures of near‐critical changes before the catastrophic incident. Huang and Jacobsson have developed alternative empirical explanations based on the length of the temporal windows.^34^, ^35^

### 3.4 Implications of Using Distributions in Science‐Policy

For a given event (e.g., the overtopping of a seawall at a specific location from a tsunami generated by an earthquake of magnitude 8.7) the canonical science‐policy elements include:

1. the magnitude, *M*, of each probable consequence, *c* ∈*C*, associated with a single specific event;
2. their cumulative probabilities (or frequencies), *F*;
3. the delays in the occurrence of consequences, δ, (e.g., δ ≤ 24 h may define prompt deaths);
4. the severity of prompt and delayed consequences, *s*_p_ and *s*_d_; and
5. the distribution of *c* ∈*C* and *s* ∈*S*, over different groups at risk, *g* ∈*G*.

**Figure 7:**
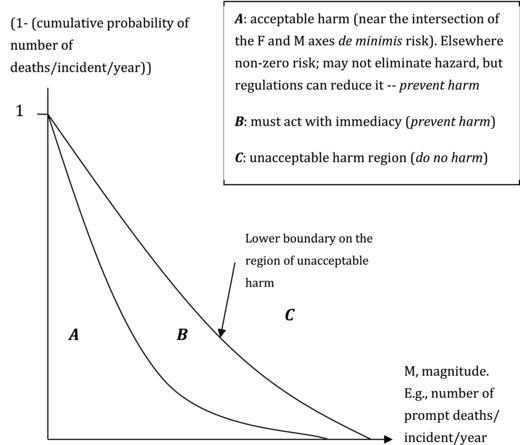
Complements of two cumulative distributions of magnitude of consequences (prompt deaths) and hypothetical acceptability regions (uncertainty about these curves omitted) for nonroutine, NR, events associated with engineered structures with known or knowable failure mechanisms.

**Figure** **7** depicts combinations of (1 − *F*) and *M* numbers used to identify alternative regions of concern to policy‐makers. These regions are bounded by the complements of the probability distributions for nonroutine accidents, but not for DKs, BSs, or Gray Swans. There is a de minimis region: it is identifiable on a case by case by legally insignificant levels of probability and magnitude, hence we do not show it. There may also be a region of resignation due to inability to act, which is case specific, in the north‐east of the depiction.

The ex ante analysis of nonroutine events, DKs, BSs, and Gray Swans suggests three main elements:

1. Mathematical—Using ordinary differential equations (ODE) or difference equations, often as systems; partial differential equations (PDF); or stochastic differential equations (SDE) to describe the dynamics of the processes generating catastrophic incidents. These models represent mechanistic phenomena and are the basis for determining bubbles, steady states, chaotic, and other trajectories. The computational methods are often numerical, rather than analytical.
2. Probabilistic or Frequentistic—For instance, consequences obtained by historical data are used to form their empirical distributions, as exemplified by the *F–M* diagrams. Data can be obtained through the analysis of past failures of key mechanical, structural or other elements (earth dams, concrete gravity dams, retaining walls, and so on). Fault‐ and event‐trees methods represent the logic of cause and effect, accounting for uncertainty through probabilities or frequencies. The frequencies of failure of individual physical components are included in the trees and propagated throughout the fault tree and event tree diagrams via various logical gates (e.g., AND, OR, and others) to characterize the top event and the ramifications of the consequences from that event. The computational method may be Monte Carlo simulations of various types. The *F*–*M* diagrams may be based on different distributions, from Poisson to stretched Pareto.
3. Statistical—Using multivariate models, simultaneous equations, lagged variables and many other expressions representing cause and effect. The computational methods may also be Monte Carlo simulations or other.

### 3.5 Distributions of Extreme Events: Overview

Regarding the temporal distributions of rare events, Benoit Mandelbrot distinguished between their being benign, wild, or having aspects of both: the CLT yields benign data; the generalized CLT governs wild ones. Some distributions include both benign and wild data. For example, the log‐normal distribution is a power law‐like distribution that, in small samples, has unstable statistical moments: the distribution is wild.

This paper deals with extreme events that fall in the right tail of what may often be a power law distribution, namely Pr(*X* ≥ *x* ∼ *cx* ^−^ *^α^*; *c*, α > 0). The coefficient α governs the thickness and shape of the tail and becomes a parameter estimated from a sample; *c* is the normalizing factor.^36^ For example, taking *f*(*x*) = *ax^k^*, where *f*(*x*) may be the frequency of avalanches and *x* their size: *k* = −1.6 over approximately four orders of magnitude of the size of the avalanches.^37^, ^38^ At the criticality, *f*_c_, a distribution function changes to a power law with a fractal exponent, *k*. For a number of policy options, the extreme values of (stationary) deviations from a policy threshold may be important. These follow the generalized Pareto distribution power law. Thus, if *y_i_* = *x_i_* − *u*_m_, where *u*_m_ is a sufficiently large threshold, then *pr*(*Y* ≤ *y_i_*) = (1 − (1 + *ξy*/σ)^−1/^ *^ξ^*) for ξ ≠ 0 or, if ξ = 0, then *pr*(*Y* ≤ *y_i_*) = (1 − exp(−*y*/σ)). Here, ξ > 0 results in a power law; ξ = 0 it is an exponential; when ξ < 0 the distribution has a finite upper bound; σ is the scale parameter. When data are truncated, analysts may use the cumulative Pareto distribution: *F*(*x*) = 1 − (α/*x*)*^β^*; *x* ≥ α; α > 0 is a lower truncation value, and β is estimated from the data. Its complement, 1 − CDF, has self‐similarity characteristics with fractal parameter β; its slope on a log scale is approximately linear over several orders of magnitude of the consequences. The tapered Pareto distribution, *y* = 1 − (α/*x*)*^β^* exp((α − *x*)/θ), lacks the self‐similarity inherent to the Pareto distribution and has a shorter right tail. Pareto distributions are heavy‐tailed relative to the Gaussian.^39^ Power laws also apply to spatial data, e.g., we exemplify circles approximating craters, for *n* = 50; *n* = 200, depicted in Figure S2 in the Supporting Information. Parameter estimation of power distributions uses cumulative rank frequency methods because very rare events, which may occur at longer intervals than the most frequent ones, affect the accuracy of the MLE.^40^ As an example of these distributions, Kagan and Schoenberg forecast a magnitude 10 seismic event associated with the tapered Pareto (TP, α = 0.67) and Pareto (*P*, α = 0.67) distributions of earthquakes from the same time series of historical data.^41^ The TP yields a return period of 1/10^436^ years, while *P* yields an ≈1/100 years return.^41^ The differences between these predictions suggests that the choice of distribution function cannot only be based on historical data alone, but should also include mechanistic (e.g., physical) processes that generate them. In summary, heavy tailed distributions combine characteristics such as:

1. The energy needed to cause the catastrophic event is small but can be amplified endogenously.
2. The dynamics of the system associated with outputs such as long‐tail distributions are similar at the macro and micro scales. Power law distributions are generated by nonlinear systems or by systems with multiplicative amplification.^24^ However, these empirical results may be statistical artifacts.^42^, ^43^, ^44^, ^45^, ^46^, ^47^, ^48^ In that case, an alternative is to use rank‐order estimators. Power law distributions include stretched, tapered, and parabolic fractal distributions in which (frequency) ∝ *n^−b^*exp(−*c*(log(rank)))^2^. A stretched distribution may be more realistic than either an exponential (*c* = 1) or other power law (*c* < 1). Stretching means that the magnitude scale is lengthened by *t* ^1/^ *^c^* and implies multiplicative events.
3. Time series may either be stationary or not. A stationary time series simplifies statistical modeling and estimation For instance, the worldwide cumulative loss from flood events over time, *t*, is characterized by *t* ^1.3^, and is not stationary.^42^ Yet, the distribution of floods or individual losses is stationary.^43^
4. Stability parameter α: an α‐stable distribution is the attractor for both the central limit theorem (CLT) and the generalized central limit theorem (GCLT) (Mantegna and Stanley).^43^ If the variance is finite, the CLT applies; if not, the GCLT applies. For example, f(*x*) = *αx* ^(^ *^α−^* ^1)^ implies that *F*(*x*) = 1 − (1/*x*)*^α^*, for *x* > 1: this distribution converges to a α‐stable distribution as *n* tends to infinity. A very large number of observations is needed to observe such convergence and hence it may not be practically observed. Stable distributions are natural limits for linear combinations of means of independent random variables. A linear combination of i.i.d. random variables results in self‐similarity. When a distribution has a very heavy right tail, its mean or variance can be infinite and it may be necessary to make those moments finite through constraints based on physical, rather than probabilistic, considerations.
5. Catastrophic incidents may cluster; their identification requires the combination of statistical methods to study autocorrelations and special statistical estimators.^42^, ^49^ Clustering may lead to events outside the characteristics of Black Swans and yield Dragon‐Kings.^41^

### 3.6 Mechanistic Aspects of Catastrophic Incidents

**Figure 8:**
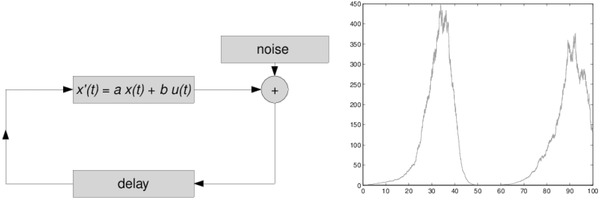
Logistic differential equation (ODE) with delay feedback, additive noise (arbitrary units) in the left panel; right panel depicts its trajectory (arbitrarily coordinates).

Mechanistic processes are often formulated through changes in the output variable, given domains such as time and space, that predict the trajectories of the magnitude of outcomes over those domains. A 1D example of deterministic process for proportional growth over time is described by an ODE: a (homogeneous) differential equation with a known initial condition (at time 0), *X_t_*_0_: d*X*/d*t* = *aX_t_*, and general solution *X_t_* = A(exp(*at*)); *A* is a constant. Its particular solution is *X_t_* = *X*(*t* = 0)exp(*at*). **Figure** **8** depicts an ODE with delay feedback and additive noise (J. Bierkens, jbierkens.nl, accessed August 3, 2015), and a trajectory generated by the logistic differential equation *X*'(*t*) = *aX*(*t*) + *bU*(*t*).

**Figure 9:**
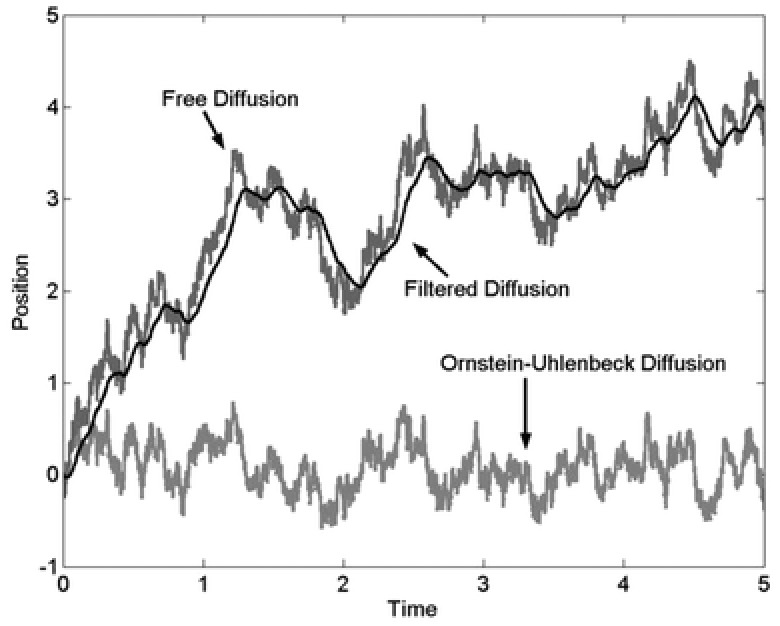
Trajectories generated by two different diffusion processes, arbitrary time and position values, and a filtered approximation to the free diffusion time series of data.

If the initial condition is random, *X*_0_(*w*), its solution is *X_t_*(*w*) = *X*_0_(*w*)exp(*at*), which depends on each individual value *w* (*w* ∈*Ω*, *Ω* = outcome space). If *a* also is a random variable, then: d*X_t_*(*w*) = (*a*d*t*+d*W_t_*(*w*))*X_t_*(*w*), with initial condition *X*_0_(*w*). More generally, we can write the stochastic differential equation, SDE: d*X_t_*(*w*) = *f_t_*(*X_t_*(*w*))d*t* + *σ_t_*(*X_t_*(*w*))d*W_t_*(*w*). This equation has a deterministic component, drift (a function of time and *X*), a random diffusion component, due to concentration differences, *σ_t_*_,_ and a noise component, d*W_t_*(*w*). Solving the first integral is straightforward; the second requires integration methods such as Ito's or Stratonovich's.^50^, ^51^ A simple example is diffusion used to predict exposure to a hazardous event such as liquid or gaseous spill.^52^ Free diffusion over time, *t*, is modeled as *X*'*_t_* = d*X_t_* = σd(*W_t_*); *W* denotes a Wiener process. Another diffusion process is Ornstein–Uhlenbeck's: d*X_t_* = *k*(μ − *X_t_*)d*t* + σd*W_t_*, which represents a physical process with an harmonic oscillator characterized by Gaussian noise; *k* is the spring constant, and μ is the spring's equilibrium value. Oscillation (or variability) is represented by (μ − *X_t_*)).^52^ **Figure** **9** depicts alternative diffusion trajectories; filtered implies the statistical fitting of a continuous function to the (free) diffusion data.

Financial catastrophes may be as important as physical catastrophes. For instance, the financial bubble and crash model of Johansen–Ledoit–Sornette^53^, ^54^ has a theoretical basis: rational expectation and herding behavior. It may be modeled as an SDE with both drift and jump, in which the stochastic component is a Wiener process *W*∼*N*(0, 1)).

When a dynamic system's model is linear, its analysis is relatively easy. However, physical reality is often nonlinear: for example, it may consist of oscillators with relaxation thresholds; in connected nonlinear systems, the suppression and control of chaos depends on couplings.^55^ Nonlinear behaviors include breaks or bumps in the right tail of the empirical distribution of the output from those systems. Additional considerations include the following.

*Liapunov exponent (coefficient)*—Clarifies the dynamics of DKs, BSs, and Gray Swans by measuring the distance between points on two or more trajectories^56^ and describes repelling and attracting characteristics of the manifold.^57^ It informs about the divergence of trajectories (or orbits) as follows: (i) when it is positive, the result is chaotic; (ii) when zero, it is a bifurcation; (iii) when negative, it is periodic. For example, the nonlinear deterministic 1D logistic map (*u_n_*_+1_ = *ru_n_*(1 − *u_n_*)) (Section 4) may have a 1‐point attractor: oscillations tend to a unique value, after some time, if the control parameter is 1 ≤ *r* ≤ 3. An example of the power spectra for the logistic map (Section IV) with two alternatives: (i) control parameters equal to 3.5, initial condition 0.8; and (ii) control parameter set at 4.0 and the same initial condition, 0.8, are depicted in Figure S1 in the Supporting Information.

*Fixed points*—Determine when trajectories are stable and, if stable, whether they are asymptotically stable. A fixed point of a mapping *F* on a set *X* is a point *x* ∈*X* such that *F*(*x*) = *x*; a fixed point is stable when it attracts nearby orbits; else, it is either unstable or there are several fixed points (Section IV).

*Correlations*—Describe coupled dynamic behaviors of variables or observations over time (or space) within a trajectory or time series of data. Long correlations (e.g., over time) have high values in the low spectral region (e.g., 0.001 Hz); higher frequencies (5 Hz) imply short correlations Specifically, short means that *f*(*t*) is proportional to *exp*(−*t*/*t*_0_) and long proportionality to *t^−k^*. Both are indicators of change for different characteristics of a system close to equilibrium. They are used to identify critical slowing down and flickering.^58^ For example, autocorrelation functions measure the slowing down in tectonic activity. The rate of change of the amplitude can be an early warning indicator of new behavior for the system leading to a catastrophic incident. For instance, an amplification by small forces can make independent events dependent.

*Emergent phenomena*—Imply self‐organizing aspects of the system likely to generate them. They consist of the autonomous ability to develop a new structure, which is wholly different from the initial one. For example, a highly connected system, or a system characterized by low diversity, has different levels resistance to change. Emergent properties cannot generally be established from analyzing single heterogeneous components (the structure of a single hand does not allow deduction of the entire human body).

*Self organizing criticality*, SOC–A dynamic system, at equilibrium with its surroundings, suddenly is at disequilibrium due to a spontaneous change.^59^, ^60^ A mechanism that exogenously adds energy may cause a relaxation leading to the catastrophic incident. A critical value separates these behaviors: typically, the log–log plot of the distribution changes from a flat to having a negative slope. Before the SOC, the distribution of failures might be exponential. At the SOC the change yields a distribution with thicker right tail, such as a power law. At the criticality, where the distribution was exponential, independent components become dependent and the distribution changes to Cauchy's. Specifically, log–log plots of coupling strength and synchronization may indicate that: (i) large coupling values imply low heterogeneity: DKs may be predictable; (ii) intermediate levels of either suggest a BS and unpredictability; (iii) low coupling strength and large heterogeneity imply incoherent behavior. SOCs suggest emergent behaviors from amplifying response through positive feedbacks or mutual microbehaviors that change both processes and nature of the outcomes.^54^ These behaviors may be due to herding (more agents than strategies leading to crowded regimens), coordination (between mechanisms), positive or delayed feedbacks, and synchronicity (increasingly stiff system).^23^

*Noise*—Sornette (2006) discusses aspects of noise, such as crackles from avalanches, which can result from heterogeneous material undergoing critical change.^42^ These events occur at different granularities; the activation of a large avalanche suggests simultaneous couplings between grains: the stronger the couplings, the larger the avalanche.^23^, ^61^ Understanding noise (as frequency) benefits from using the power spectral density, PSD: the Fourier transform of the autocorrelation function. Oscillations in the low spectral region (e.g., 0.001 Hz) characterize long time scale correlations; high values the high spectral region (e.g., 5 Hz) suggest short correlations. The PSD can be formulated as log(power) = g(log(frequency)) using frequencies (in Hz) and power (Joules/second): PSD = 1/*f^β^*; β ≥ 0. Specifically, β = 0 implies white noise (equal power at any frequency); β = −1 is pink noise; β = −2 is brown noise. White noise is flat; pink noise has slope of 1/*f* implying self‐similarity and scale invariance at all frequencies. Other aspects of noise include^42^:

1. Pops: Multiple infrequent noise.
2. Snaps: Single independent event.
3. Shots: Sporadic bursts.
4. Crackles: Random in amplitude and time, are scale independent and show the same irregularities and amplitudes regardless of scale.
5. Flickering: Change in the system's state, determined from a time series, results in a change from one set of realizations to a markedly different set.

## 4 Example: Logistic 1D map

We use http://demonstrations.wolfram.com/TheLogisticDifferenceEquation/ to exemplify how the 1D nonlinear, dynamic, autonomous model—the logistic difference equation (or map) *u_n_*_+1_ = *ru_n_*(1 − *u_n_*)—yields policy information through its alternative trajectories. Consider values of u ∈ U: *f*(*u_t_*) which change over time.^62^ The solutions run from the predictable to the chaotic and occur through numerically changing the values of the control parameter, *r*. The example shows alternative solutions of the logistic model due to the choice of values for: the control parameter, initial conditions; and time delays. The details are shown in Figures S3, S3a,b and S4, S4a,b in the Supporting Information. The following observations suggest how science‐policy benefits from modeling (**Table** **2**).

**Figure 10:**
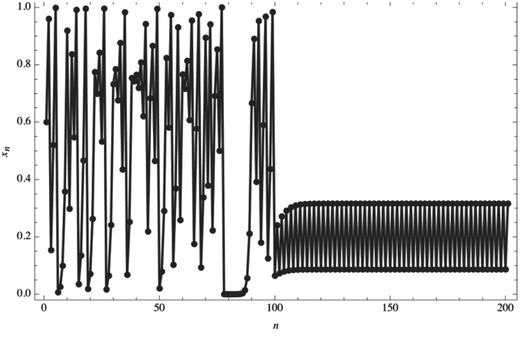
Controlling chaos on the logistic map.

1. Trajectories tend to: (i) a stable point, (ii) a cycle, or (iii) are chaotic The evolution of the system depends on the value of *r* and on the system's initial conditions. Each solution has a corresponding cobweb diagram: the green line intersects back and forth between the graphs, beginning at (*u*_0_, 0). Each intersection of the green line and the red parabola represents a value of *u_n_*; the solution may converge to a single point, oscillates, or be unpredictable. The equilibrium values for *u_n_* determine when a long‐term solution is predictable. If 0 < *r* ≤ 1, there is an asymptotically stable value. For 1 < *r* < 3, the solutions converge to *u* = (*r* − 1)/*r*. For *r* ≥ 3, solutions do not converge to a fixed point, except when *u_n_* = (*r* − 1)/*r* exactly for some *N*, where *u_n_* = (*r* − 1)/*r* for all *n* >*N*.
2. Practically, it may be implausible to use a fixed initial condition; this simplification may be relaxed by introducing a constant delay τ. The solution of the nonlinear differential equation, with scalar delay, d*x*/d*t* = *ax*(*t*)(1 − *x*(*t − τ*)) is depicted in blue, and for the differential equation without delay (*t* = 0), in red (Delay Logistic Equation, http://demonstrations.wolfram.com/DelayLogisticEquation/) **Table** **3**, Supporting Information. For *aτ* < 1/*e*, the solutions are monotonic; for 1/*e* < *aτ* < π/2, they are oscillatory and asymptotically approach *x* = 1. For *a* > π/2, the solutions approach a limit cycle. For delay τ = 1, no delay (τ = 0) models, and *a* = 1 we obtain solutions and phase diagrams (Table S3, Supporting Information.)
3. Chaotic behavior may be controlled through a specific policy intervention. For instance, applying a periodic proportional pulse (pulse = 0.1, applied at period 2 during the iteration process), given the equation's control set to *r* = 4, which yields the results depicted in **Figure** **10**. Although the solution from n = 100 still oscillates, the oscillations are predictable relative to the interval (0, <100).

**Table 2:**
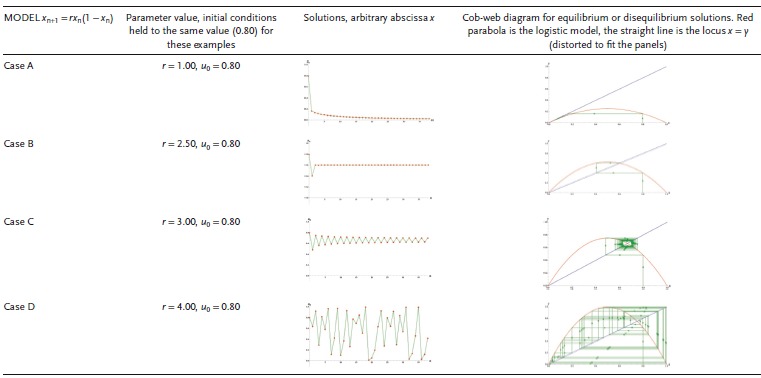
Logistic map depicting different behaviors resulting from alternative values of the control parameter, *r*

**Table 3:** Individual expert opinions on premises, propositions, and results lead to aggregate judgments and the discursive paradox

Expert's number	Proposition, *p*	If (*p* AND *q*) ↔ *r*	Therefore proposition *r* is:	Comments
1	T	T	T	NA
2	T	F	F	NA
3	F	T	F	NA
Aggregate Judgment	T	T	F	Majority rule
Uncertainty is not included	T: true; F: false	↔: if and only if (joint necessary and sufficient conditions) for r.	NA	References: Dietrich and List (2012)

## 5 Aggregation of Scientific Expert Opinions

**Figure 11:**
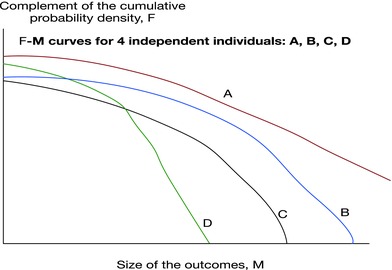
Hypothetical complements of subjective cumulative distributions, (equivalent to four *F*–*M* diagrams). The subjective probability axis (probability [0, 1]) and magnitude (*M*), for the four experts is elicited and based on their knowledge, rather than being fitted to historical or experimental data as would be the case for *F*–*M* diagrams.

Dealing with predictions suggests a critical role for experts' judgments and beliefs.^63^ A simple way to quantify an expert's opinion is to let the person be assessed and provide the overall uncertainty about the implication of the risky event of concern. Each expert view is elicited (e.g., using probability wheel methods) to quantitatively describe the relationship between sizes of the consequences and their probability—for a given event familiar to the experts. **Figure** **11** depicts continuous complements of the cumulative distribution functions that summarize four hypothetical experts' elicitations. These experts do not produce historical Frequency‐Magnitude distributions because they are encoding personal beliefs through probabilities as subjective degrees of belief.

For experts B, C and D the complement of their cumulative *F*–*M* distributions has a finite maximum magnitude consequence and convergence to this bound is rapid. However, there is no such upper bound for expert A. Her description is a power‐law: a scale free distribution over several orders of magnitude of the consequences (roughly, the linear portion of the curve), unlike the judgments of others. These three results may lull policy‐makers into a false sense security: the majority strongly supports an upper bound. However, judgments may be affected by biases; A's minority view may be discarded for reasons that range from unwillingness to accept a truly catastrophic event, to A being an outlier (the *Cassandra* effect).

### 5.1 Aggregating Individual Scientific Beliefs

Science‐policy inevitably confronts several alternative analyses of catastrophic incidents, each of which has different visions of reality, assumptions, models, uncertainty, and results. National panels and advisory boards use agreement to inform policy. Aggregating different opinions requires formal methods through which a set of individual assumptions, models, data sets, and results are assessed and can be verified by others who have a stake in the matter. A limited search of the government web sites did not disclose the details needed to understand how agreement (or consensus) is reached. Thus, it is difficult to determine: (i) voting criteria (e.g., majority), (ii) how votes are counted and weighted, (iii) the details of method used to aggregate multiple expert opinions, and (iv) the divergences between individual expert opinions regarding each expert's assumptions, model, and results. Table 3 lists the elements of individual scientific beliefs of three experts assessing scientific evidence and aggregates their preferences via simple majority rule. Experts are assumed to be independent, demonstrably qualified, and have no vested interest in the outcome of their assessments. We exemplify these ideas through an example consisting of Boolean (true (T), or False(F)) states, a proposition (assumption) *p*, logical connection (AND), a single If… Then causation, and a result *r*. Agreement on the result is false under the majority rule, while the same rule makes both the assumptions and model (IF …, THEN …) true (Table 3).

Principles for aggregating individual opinions include as unanimity, anonymity, monotonicity, and systematicity. Critically for science‐policy, majority or other rule for aggregating multiple stakeholders' assessments, are demonstrably dictatorial unless one or more of these principles are relaxed. Yet, they are essential requirements for selecting between alternative judgments.

## 6 Conclusion

We have discussed salient aspects of catastrophe modeling needed to inform science‐policy. We conclude that the distributions of catastrophic consequences from seismic, volcanic, and other natural and human catastrophic incidents (or events) may have either thin or fat tails, depending on their generating mechanisms. Their modeling rests on theoretical and empirical foundations. Regarding the empirical aspects, nonroutine events are characterized by thin‐tailed distributions. However, catastrophic incidents that include extremely large consequences, such as Black Swans, and Gray Swans, are often associated with fractal distributions with fat right tails, although Dragon‐Kings are not. The mechanisms generating catastrophic incidents can be ordinary differential equations, ODEs, and partial differential equations, PDEs. These are deterministic; uncertainty may be accounted using stochastic differential equations, SDEs. The models' solutions (e.g., steady‐state, chaotic or other, including the neighborhood of criticalities as precursor signals for the catastrophe of concern), and aspects of the distributions that characterize these events inform science‐policy ex ante of the catastrophic incident. Policy makers can then opt for precautionary or preventive actions, including doing nothing. We conclude that the analysis of catastrophic incidents should account for:

1. Knowledge about the dynamical and probabilistic aspects of the system likely to generate a catastrophe should be described theoretically and empirically through formal models (such as differential or difference equations), and include stochastic processes.
2. Empirical analysis of time series and spatial data regarding output and input variables, and account for short‐ and long‐term correlations between the data that affect the upper tail of their distributions and hence the risk profiles (e.g., *F*–*M* curves).
3. Probability distributions should be based on the mechanisms likely to generate the adverse events of concern and thus include distributions characterized by thick tails, rather than assume asymptotic behaviors' leading to rapid convergence to a normal distribution,
4. Types of noise, e.g., bubbling, and implications of noise on the predictability of outcomes, identify the nature of catastrophes and give some warning of their impending occurrence,
5. Internal perturbations to the system likely to generate a catastrophe affects the probability of occurrence, and the
6. Sensitivity of the dynamical systems that model catastrophes to changes in their initial conditions, time delays, feedbacks, and other physical conditions.

Corrective actions imply cost‐effectiveness and cost‐benefit analyses that can be readily included in these assessments. Our last conclusion regards scientific choices made by aggregating experts' judgments. Their aggregation should be based on principles such as unanimity, anonymity, monotonicity, and systematicity to obtain a democratic outcome, and consists of aggregating over three canonical elements of the discourse: assumptions, models and results, using a decision rule such as simple majority. Yet, impossibility theorems confirm that only the dictatorial solution meets these principles, unless the set of principles is reduced.

## Conflict of Interest

The authors declare no conflict of interest.

## Supporting information

Supplementary
