# Supplementary material for: Assessing Catastrophes—Dragon‐Kings, Black, and Gray Swans—for Science‐Policy
Source: Glob Chall. 2017 Jul 28;1(6):1700021. doi: 10.1002/gch2.201700021 (PMC6607139; doi:10.1002/gch2.201700021)
Supplement: Supplementary file 1 — Supplementary [file GCH2-1-1700021-s001.pdf]

Copyright WILEY-VCH Verlag GmbH & Co. KGaA, 69469 Weinheim, Germany, 2017.

Global Challenges

Open Access

Supporting Information

for *Global Challenges*, DOI: 10.1002/gch2. 201700021

Assessing Catastrophes—Dragon-Kings, Black, and Gray Swans—for Science-Policy

Paolo F. Ricci and Hua-Xia Sheng*

Table S1, Cases of Probability Distributions of Rare Events

Table S2 Summary of Information Embedding High Dimensionality Dynamics of Time Series or Sequence of Data in a Recurrence Plot

Table S3, Delayed Differential Equation versus Ordinary Differential Equation, Resulting Trajectories and Phase Diagrams

Figure S1 Examples of Power Spectra for their Time Series, Associated with of the Logistic Map

Figure S2 Spatial Power Law Distributions of Cratering Data

Figure S3, Time Series Generated by the Logistic Map (Control Parameter = *3.5*)

Figure S3a, Recurrence Matrix of the Logistic Map with Control Parameter Value Set to *3.5* and Threshold set at $\varepsilon = 0.05$

Figure S3b, Recurrence Network Associated with the Logistic Map with Control Parameter = *3.5*

Figure S4, Recurrence-Based Representations of the Logistic Map（control parameter = *4.0*）

Figure S4a, Recurrence Matrix of Logistic Map (control parameter = *4.0*)

Figure S4b, Recurrence Network of Logistic Map (control parameter = *4.0*)

Table S1, Cases of Probability Distributions of Rare Events (from Mandelbrodt (1997)[14] and Sornette (2006)[15])

Cases	Characteristics	Representative Distributions	Results	Examples	Comments
Benign, with some issues	Rapid convergence of sums of normalized i.i.d. RVs to the Gaussian, via the CLT, (e.g., speed of convergence measured by the ratio of third moment to cubed standard deviation).	Normalized Gaussian pdf; finite variance, stable under convolution (addition of i.i.d. RVs). Mean and variance uniquely identify the pdf.	Thin tails. Relatively large magnitudes are extremely small (e.g., > 5 sigma implies ~$6*10^{-7}$ probability.	Thermal noise; coding DNA sequences for benign and non-coding sequences for long correlations. Diffusion enhanced depending on mixing.	The Gaussian pdf is the "attractor"; i.i.d. assumptions can fail due to correlations (first i in i.i.d.).
Slow	Slow convergence to a finite limit, not necessarily Gaussian. Large (and extremely large) deviations theory extends the *benign* case to non-identically distributed RVs.	Log-normal (LN), Weibul; Pareto-like; Log Normal is stable under logarithmic addition of RVs. Mean and variance do not uniquely identify the LN pdf.	Long upper tail, eventual convergence, can be similar to a (1/f) power law for a large interval, slow convergence (for large σ_X).	Western US coastline; earthquake inducing deformations; turbulent flows, fragmentations.	Heavy tails and stretched exponential distributions generated by a single very large x-value. Quasi-stable stretched exponential pdf.
Wild	1/f noise, extremely long cycles with convergence as generalized CLT.	Le'vy, e.g., Cauchy with infinite variance; stable distributions.	Thick (lower) and upper tails; may have either rapid or slow convergence.	Large floods; financial catastrophes.	Symmetric or asymmetric. The attractor is Le'vy's law (generalized CLT).
Mixed	Different forms of noise: from white to brown.	Different states, but not mixtures of distributions.	Thick tails.	Cross-over regimens.	Changes from Gaussian in the center ($x < (N\ln N)^{0.5}$ to power-law in the tails $x >> (N\ln N)^{0.5}$.

Note: X is a random variable (RV) with x a probabilistic realization; pdf is probability distribution function; N is the number of random variables; i.i.d. = independent, identically distributed. CLT is Central Limit Theorem. Following Sornette (2006), for a random variable X (continuous values of X labeled x), the variance, Var(X), may not exist when a tail decays slower than x^{-3}, x being sufficiently large. If f(x) = C/(|x|^(1+μ)), $x \rightarrow \pm\infty$ and $\mu \leq 2$, then Var(X) does not exist.

Table S2 summarizes different aspects of time series or sequence of data and their interpretation in recurrence plot analysis.

Table S2, Summary of Information Embedding High Dimensionality Dynamics of Time Series or Sequence of Data in a Recurrence Plot

Aspects of time series or sequence	Characteristics	Implications	Plot Configurations	Comments
Homogeneity	Stationary process, autonomous (independent of time).	Random time series or sequence of observations.	Randomly dispersed points over the plane.	Relaxation time short relative to length of observations.
Periodicity	Seasons or cycles, time dependent,	Periodic recurrences; multiple harmonics, possible.	Diagonal lines, checkerboard; quasi-periodicities identified by different distances between diagonal lines.	Recurrence is identified by the distance between the diagonal lines running SW to NE. Chaotic behaviors identifiable by diagonals and proximate isolated points.
Drift	Non-stationary process, linear trend in the time series.	Generally increasing or decreasing values.	Density of dots higher in North-East and South-West.	Darker cluster of points localized in parts of the quadrants.
Sharp changes (e.g., steps)	Sudden changes in the dynamics of the system.	Extreme occurrences readily identifiable.	Light and white bands or areas.	Multiple, abrupt, or rare events.
Large oscillations	Lack of correlations.	Possible anti-correlations.	Single, isolated points.	None.
Epochs	Similar epochal dynamics.	Different physical properties.	Long bowed lines.	Eventually changing dynamics.

Adapted and modified from http://www.recurrence-plot.tk/glance.php;

Examples of alternative solutions obtained using delayed differential equation and ordinary differential equations, including their associated phase diagrams depicted in Table S3.

Table S3, Delayed Differential Equation versus Ordinary Differential Equation, Resulting Trajectories and Phase Diagrams (blue, red plots respectively)

**MODELS**<br>**dx/dt = = ax(t)(1-x(t-τ))**<br>**in blue and**<br>**dx/dt = ax(1-x(t)) in red**	**PARAMETERS:**<br>**delay and 1.00 control for blue plots; control only (τ = 0) for red plots**	**SOLUTIONS:**<br>**Trajectories for delayed and non-delayed differential equations, blue and red, respectively**	**PHASE DIAGRAMS:**<br>**Blue and red plots for delayed and non-delayed differential equations**
Case A1	τ = 1.00, a = 1.00	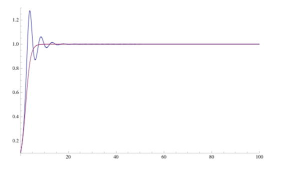	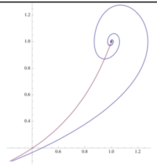
Case A2	τ = 1.00, a = 1.50,	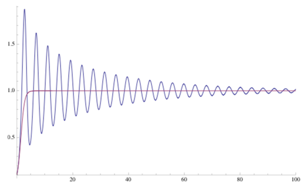	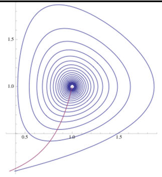
Case A3	τ = 1.50, a = 2.00	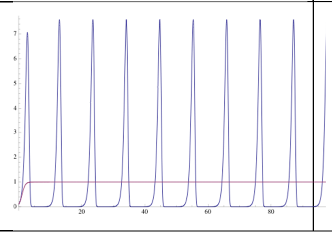	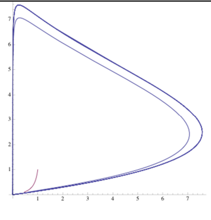

Figure S1 depicts two time series and their power spectrum generated by the 1-D logistic map used throughout this paper.

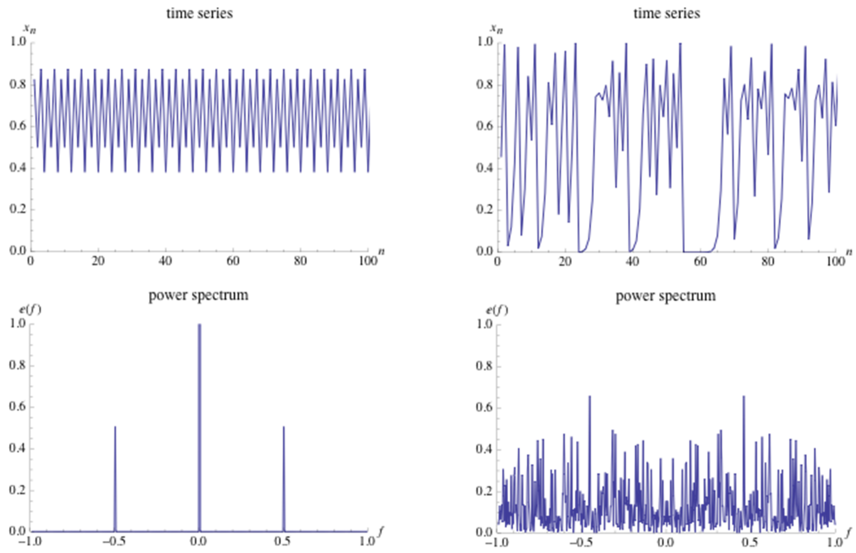

Figure S1，Examples of Power Spectra for their Time Series, Associated with of the Logistic Map

（http://demonstrations.wolfram.com/PowerSpectrumOfTheLogisticMap/）

Figure S2 depicts how circles approximating cratering result in spatial power law, for different sample sizes equal to *n = 50*; *n = 200*, respectively.

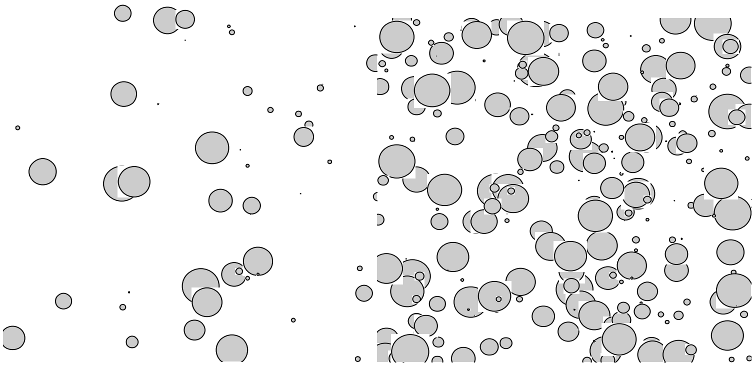

Figure S2, Spatial Power Law Distributions of Cratering Data (n = 50; n= 200),

http://demonstrations.wolfram.com/RandomCirclesWithPowerLawSizes/

For the time series depicted in Figure S3

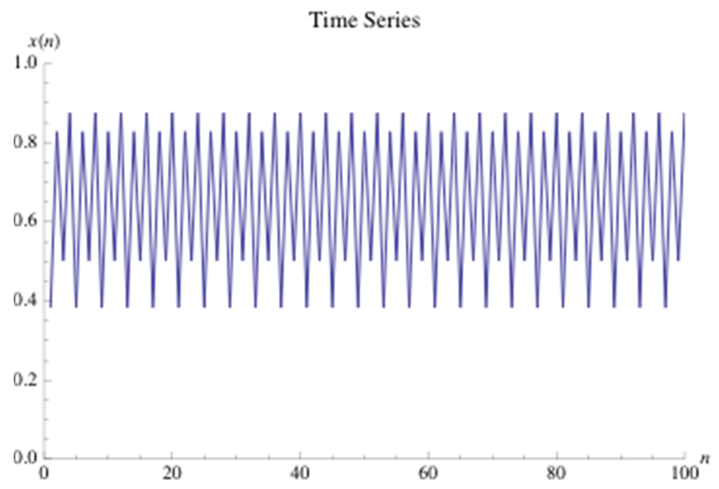

Figure S3, Time Series Generated by the Logistic Map (Control Parameter = ***3.5*****)**

Defining two time steps to be recurrent if and only if their distance is smaller than a threshold, ε [34], which we set to *0.05* we obtain the recurrence matrix depicted in Figure S3a:

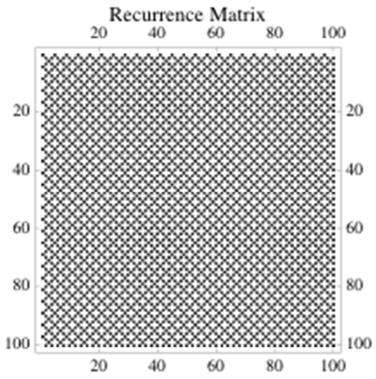

Figure S3a，　Recurrence Matrix of the Logistic Map with Control Parameter Value Set to 3.5 and Threshold set at $\varepsilon = 0.05$

For 100 iterations, each of which is a node of the recurrence network, *recurrence* in the phase space is either a link or a black dot. The network associated with this example is depicted in Figure S3b.

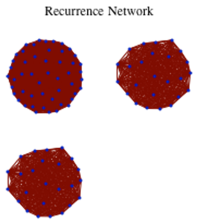

Figure S3b，Recurrence Network Associated with the Logistic Map with Control Parameter = 3.5

This network identifies a complete separation of clusters. An entirely new configuration, obtained by changing the control parameter to *4.0*, generates the time series:

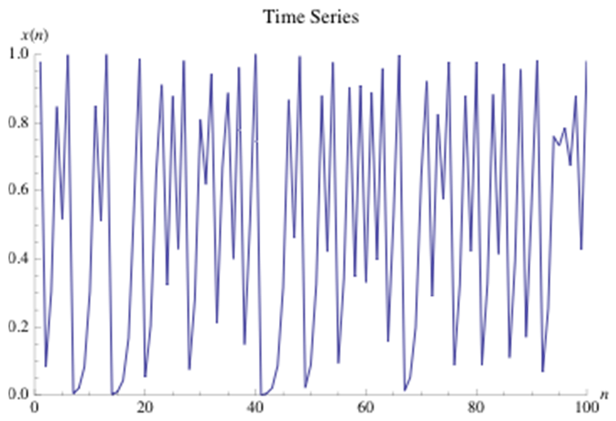

Figure S4，Recurrence-Based Representations of the Logistic Map（control parameter = *4.0*）

Its recurrence matrix is:

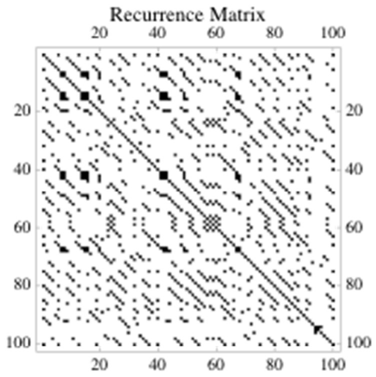

Figure S4a，Recurrence Matrix of Logistic Map (control parameter = *4.0*)

The corresponding network is now weakly connect, including a disconnection, as depicted in Figure S4b.

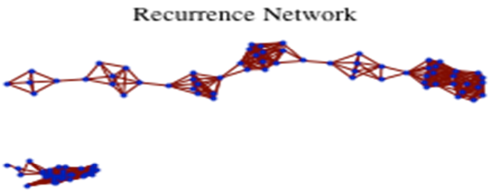

Figure S4b，Recurrence Network of Logistic Map (control parameter = *4.0*)
